# Slaughter of Pregnant Cattle in Denmark: Prevalence, Gestational Age, and Reasons

**DOI:** 10.3390/ani9070392

**Published:** 2019-06-27

**Authors:** Søren Saxmose Nielsen, Peter Sandøe, Stine Ulrich Kjølsted, Jørgen Steen Agerholm

**Affiliations:** 1 Department of Veterinary and Animal Sciences, University of Copenhagen, 1870 Frederiksberg C, Denmark; 2 Department of Food and Resource Economics, University of Copenhagen, 1870 Frederiksberg C, Denmark; 3 Department of Veterinary Clinical Sciences, University of Copenhagen, 2630 Taastrup, Denmark

**Keywords:** cow, Danish, ethics, gestation length, pregnancy, slaughter

## Abstract

**Simple Summary:**

Cattle farmers are in a dilemma when they have to decide if an animal is fit for continued production and can endure another lactation or a pregnancy, or if slaughtering the animal is a better solution despite the animal being pregnant. We studied the prevalence of pregnant cattle at a Danish abattoir and did follow-up interviews of the farmers, asking the reasons for the slaughter of the specific animals, and queried their ethical deliberations. Many pregnant cattle were slaughtered, and the decision to do so was often health-related in dairy herds and production-related in non-dairy herds. Farmers were often aware of the ethical dilemma and considered it better to slaughter the pregnant cow instead of letting her endure another lactation in a stressful environment.

**Abstract:**

The slaughter of pregnant cattle gives rise to ethical controversy. We estimated the prevalence of pregnant cattle, elucidated the reasons for their slaughter, and in light of our findings, discussed the ethics of sending pregnant cattle for slaughter. Among 825 female cattle >353 days of age admitted to a Danish abattoir, 187 (23%) were found to be pregnant. There was no apparent difference in the proportion of pregnant animals between dairy and non-dairy cattle. “Health”-related slaughter was most frequent in dairy herds (70%), whereas “production”-related slaughter was most frequent in non-dairy herds (63%). While many farmers considered it unethical to slaughter pregnant cows without a good reason for doing so, many dairy farmers identified animal welfare as an important parameter in the decision, which was typically when the general condition of the cow or heifer would make it difficult for her to pass through calving and subsequent lactation. The many pregnant animals sent for slaughter were often the result of deliberate choices. Non-dairy farmers often kept a bull with their female cattle, and in many instances, this resulted in the mating of cattle intended for slaughter. Although considered ethically problematic by many dairy farmers, the slaughter of pregnant dairy cattle was often considered better for the cow compared to a stressful lactation period.

## 1. Introduction

Reproduction is an essential part of the beef and dairy production cycle. In beef production, this results in calves for veal and beef production, and in dairy production, it results in lactation for the cow, heifers for replacement, as well as calves for veal production. However, in some cases, pregnant animals are slaughtered. The most recent studies were conducted in 2011 and were based on questionnaire data from abattoirs in Germany [1,2]. Studies with specific recordings on the individual level include older studies: a prevalence of 26% pregnant cattle at slaughter (*n* = 1000) was recorded in the United States before 1954 [3], and in the United Kingdom, prevalences of 30% (*n* = 1032 dairy cows), 10% (*n* = 100), 23% (*n* = 1885), and 23% (*n* = 2502 cows) pregnant cattle at slaughter were recorded between 1959 and 1961 [4], before 1970 [5], in 1974–1975 [6], and in 1991 [7], respectively. Furthermore, a study in Australia in 1973 has estimated a prevalence of 63% pregnant heifers and cows slaughtered among 7495 extensively farmed beef cattle [8]).

Concerns have been raised regarding the ability of foetuses to experience pain or other negative affect based on hormone values, behaviour, heart rate, and electroencephalograms, in the last trimester of gestation [1,2,9]. However, the general consensus is that these concerns are unfounded [10], since the above-mentioned factors are not seen as indicators of pain, because pain requires actual and not just potential sentience, and according to the state-of-the-art literature, this does not apply to a bovine foetus in an unopened uterus at an abattoir [11,12,13,14,15,16] (see however, Campbell et al., 2014 [17] for a discussion of uncertainties relating to the conclusions of this literature). Nevertheless, in 2017, Germany banned the slaughter of pregnant cattle during the last trimester of gestation to protect the foetus [18]. The transport and slaughter of pregnant cattle during the last 10^th^ of gestation is also prohibited within the European Union to protect the dams against stress and injuries during transportation, and because water and energy requirements increase significantly in late pregnancy [19].

Cattle are pregnant for around 281 days, and in the period from an established pregnancy to calving, the animal can be susceptible to disease, injury, or other unforeseen conditions that makes the farmer decide to slaughter the animal, even when they would not slaughter pregnant cattle. Furthermore, to avoid oestrus-related behaviour, it is common in Denmark to inseminate eligible dairy cows until they become pregnant even though they are designated for slaughter (P. Raundal, SEGES, Aarhus N, Denmark, personal communication).

Reasons for sending pregnant cows for slaughter include presumed infertility (cattle erroneously considered to be non-pregnant), low production, and mastitis [7,20]. However, no recent data are reported, and the two studies mentioned above focused only on dairy cattle rather than cattle kept in an extensive production system, which has a different production cycle. A recent systematic literature review followed by expert knowledge elicitation [21] characterised the main reasons for slaughter of pregnant cattle as economic (e.g., low productivity), management-related (e.g., false-negative pregnancy diagnoses or pregnant cows being calmer than non-pregnant cows and thus resulting in fewer injuries in the herd), and health and welfare related (e.g., lameness, mastitis, disease control) [10].

To our knowledge, there are no studies that reflect the thoughts and deliberations of farmers sending pregnant cattle for slaughter. The scientific and legislative focus has been on protecting the pregnant animals during transportation, human exposure to oestradiol-17β or progesteron [1], and the disputed foetal pain [2,10], but the balance between keeping a pregnant but potentially diseased or injured animal in an intensive production environment versus sending it to slaughter has not been assessed [10].

Therefore, the aims of this study were to assess: (a) the prevalence of pregnant cattle at slaughter in different production systems in Denmark; (b) if the farmers were aware that they send pregnant cattle to slaughter; (c) the reasons farmers give for sending pregnant cattle for slaughter; (d) their ethical considerations when sending pregnant cattle for slaughter; and (e) if the practice of sending pregnant cattle for slaughter reflects a lack of care from Danish farmers or is based on concerns for animal welfare or other ethical reasons.

## 2. Materials and Methods

### 2.1. Subsection

A Danish cattle abattoir that receives approximately 20% of the 220,000 cattle slaughtered in Denmark annually was visited from 16 to 19 October 2017. This abattoir has no specific requirements for the cattle admitted for slaughter except for those laid down in the legislation of the European Union, and both sexes of dairy and beef cattle are slaughtered here. Two assessors (a veterinarian (Anne Marie Michelsen) and a final year veterinary student (S.U.K.)) were present at the abattoir for the entire period, and one additional assessor (J.S.A.) was present during the start-up of data collection. All the cattle admitted to the abattoir in the specified period had their gender and pregnancy status assessed. The pregnancy status was assessed by direct visual inspection and palpation of the uterus at the station where meat inspection is performed. Depending on the estimated size of the foetus, the pregnant uterus was either sampled unopened (for small-to-medium-sized foetuses), or the uterus was opened and the foetus was extracted (for large foetuses). We expected to be able to observe pregnancies >21 days into gestation. Foetuses were measured 1–3 h after slaughter of the cow or heifer. The foetometric data recorded included head length (HL, mm), head width (HW, mm), crown-rump length (CRL, cm) and body weight (BW, kg) according to a previously described protocol [22]. In addition, the official animal identification number of the dam was recorded.

### 2.2. Foetal Age Assessment

The age (in days) of the foetuses was subsequently assessed using an estimator previously described [22], which has an estimated accuracy of +/− 11 days, but stratified by Jersey (J) and non-Jersey (NJ) with the following equations:

(1) $$\begin{array}{c} Age_{J}=47.26+1.26\times HW-0.01\times HL+0.56\times CRL+477\times BW+544\times BW^{2}\\ +579\times BW^{3}+289\times BW^{4}+116\times BW^{5} \end{array}$$

(2) $$\begin{array}{c} Age_{NJ}=34.39+0.38\times HW+0.47\times HL+0.78\times CRL-56.4\times BW+40.6\times BW^{2}\\ +3.4\times BW^{3}-5.2\times BW^{4}-2.6\times BW^{5} \end{array}$$

### 2.3. Telephone Survey with Farmers

Ethical approval was obtained from the SCIENCE-SUND Research Ethics Committee, University of Copenhagen (journal no. 504-0037/18-5000). The animal identification number from the official Central Husbandry Register (CHR) of the Danish Veterinary and Food Administration (available at www.chr.fvst.dk) was used to retrieve information about the farmer’s identity, herd type, breed, and parity of the dam. The farmer information was subsequently used to retrieve telephone numbers from official databases with Google search (www.google.dk) using the name of the holding as obtained from the CHR. Then, all the farmers with pregnant animals recorded were contacted via telephone by one of the authors (S.S.N.). Each farmer was contacted for the first time within 1–2 days of the slaughter of the pregnant animal. After a minimum of 10 failed attempts at least 1 h apart, and on at least four different days over the following week, efforts to contact the farmer were abandoned. One farmer was not contacted to avoid interference with a legal process, as the official veterinarian at the abattoir had recorded the dam to be more than the legally permitted 90% threshold into gestation.

The telephone survey was carried out by one person (S.S.N.), and the answers that were provided by the farmers were recorded on paper during the survey—all the questions were in Danish. Each farmer was first provided with background information, including information about the purpose of the study, and was informed that all the information would be kept anonymous and that their given consent could be revoked at any time. The farmers were also informed that they could terminate the interview and state that they did not want to share any further information at any time. Lastly, they were informed that herd-specific information would not be shared with the veterinary authorities.

During the telephone survey, each farmer was asked:

- if he/she knew that the specific animal was pregnant (yes or no);
- if he/she was the one who made the decision about the slaughter (and if not: if he/she could provide details of the relevant person—in these cases, the interview ended and that person was contacted instead);
- if he/she could provide the reason for slaughter of the individual animal within the categories: (1) health problems (such as mastitis or lameness), (2) production-related issues (such as low milk yield, repeated inseminations before the established pregnancy), (3) old age, or (4) acute unplanned slaughter;
- to share his or her attitude towards sending pregnant cattle to slaughter and provide other views on the topic.

The recorded attitudes and views were stratified by herd type (dairy or non-dairy) and estimated month of gestation, and were summarised to reveal the main points for understanding why farmers slaughter pregnant cattle. A dairy herd was defined as a herd from which milk had been delivered to a commercial dairy plant within the study period, and all the remaining herds were classed as non-dairy herds. The non-dairy herds included a mixture of veal and beef producers, producers with suckler cows, and small holders with no commercial cattle production.

### 2.4. Statistics

The prevalence of pregnant cattle was estimated as the proportion of female cattle recorded as pregnant. The age distribution of the slaughtered female cattle was then described, and parity was recorded as heifers (parity = 0, no previous calving) or cows (parity >0). Furthermore, the age of the youngest recorded pregnant heifer was used to exclude all the females below this age, and a “corrected apparent prevalence” was then estimated. This age-corrected apparent prevalence was estimated overall and stratified by production system (dairy and non-dairy) and parity level. The corrected apparent prevalence was also estimated for different gestation intervals. The stratified prevalences were compared using the Pearson Χ^2^-test, and the relative risk was calculated for statistically different (*p* < 0.05) scenarios. Then, the prevalence of farmers stating that they were aware that the animal sent to slaughter was pregnant was estimated. Lastly, the reasons for sending pregnant cattle for slaughtered were calculated, stratified by production system.

## 3. Results

**Figure 1:**
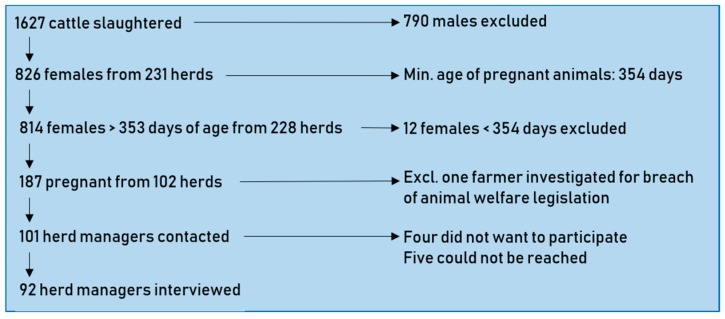
Number of animals, herds, and herd managers resulting from the various assessment steps of cattle slaughtered and assessed for pregnancy at a major Danish abattoir from 16–19 October 2017.

A total of 1627 cattle were slaughtered, including 837 females from 231 herds. Of these, 198 (24%) animals were recorded as pregnant, the youngest of which was 354 days old, while the youngest slaughtered animal was 242 days old. The corrected apparent prevalence was calculated based on animals >353 days of age, thereby excluding 12 females. A summary of the included and excluded subjects is illustrated in Figure 1. A total of 187 (23%; 95% confidence interval (C.I.): 20–26%) female cattle >354 days were pregnant. Among the pregnant cattle, 28% were in the first third of gestation, 49% were in the middle third of gestation, and 22% were in the last third of gestation. Despite the legal ban on the transport of late-term pregnant cattle, 0.4% (95% C.I.: 0.1–1%) were more than 90 (252 days) into gestation. The stratified prevalences are given in Table 1.

There was little overall difference between the prevalence of pregnant animals in the production systems (*p* = 0.36), but in non-dairy herds, the risk of being pregnant was more than two times higher in cows compared to heifers (relative risk: 2.4; 95% C.I.: 1.5–3.9). Heifers were more prevalent among non-dairy cattle, comprising 58% of the slaughtered female non-dairy cattle, and only 16% of the female dairy cattle. We defined heifers as female cattle that had not previously given birth to a calf.

The 187 slaughtered pregnant cattle originated from 102 herds, all but one of which were contacted for inclusion in the telephone survey. A total of 92 herd managers responded—61 from dairy herds and 31 from non-dairy herds—while four did not want to participate, and five could not be contacted (Figure 1), giving a response rate of 91%. Of the dairy managers, 90% (*n* = 55) said that they knew their cattle were pregnant, whereas only 65% (*n* = 20) of the non-dairy managers were aware that the cattle sent for slaughter were pregnant.

Reasons for sending pregnant animals for slaughter were provided for 168 of the 187 animals, with 3% (*n* = 5) slaughtered due to old age, 48% (*n* = 80) for reasons related to health, and 49% (*n* = 83) for reasons related to production. When only assessing pregnancy in the last two trimesters of gestation (>93 days pregnant), 30% (*n* = 17) of dairy managers used “production” as a reason for slaughter, while 70% (*n* = 39) stated health problems to be the motivation (Table 2). For the 24 non-dairy cattle >93 days pregnant, 15 (63%) were slaughtered for production-related reasons, six (25%) due to health, and one (5%) due to old age. None of the herd managers had sent cattle more than an estimated 186 days into gestation to slaughter; therefore, the data were slightly skewed towards producers sending cattle to slaughter earlier in gestation. No information on the reasons for slaughter was collected from herds sending non-pregnant animals for slaughter.

All the specific comments given by each herd manager are summarised in Table A1 for dairy managers and Table A2 for non-dairy managers. Many dairy cattle farmers identified a dilemma between deciding to send a pregnant animal for slaughter and keeping a cow that was diseased, injured, or deemed unlikely to manage an additional lactation. Several also stated that they did not slaughter pregnant animals, while evidently still doing so, even in late gestation. However, in several of those cases, the pregnancies were estimated to be further progressed based on inspection of the foetuses compared to what the farmers stated. Several farmers were also regretful that they had slaughtered a pregnant animal, but the dilemma mentioned above drove them to choose this option (Table A1). Non-dairy cattle managers mentioned better growth, better body condition, and calmer animals as reasons for inseminating female cattle. However, in many cases, pregnancies were the result of natural mating as heifers, cows, and bulls were kept together—partly because they could not be kept separate, partly because it resulted in a more natural environment for the animals, and partly because it provided a calmer herd. In some cases, the farmers were not aware that the animals were pregnant because the animals were not very old (early yearlings) or because the bulls were not expected to be fertile (pubertal) (Table A2).

## 4. Discussion

We found a pregnancy prevalence of 23% among heifers and cows, with no apparent difference between dairy and non-dairy cattle. Pregnant non-dairy cattle were mostly cows (38%, compared to 16% of heifers), while pregnant dairy cattle were both cows (23%) and heifers (20%). Dairy cattle managers were mostly aware (90%) that their cattle were pregnant when sent for slaughter, while only 65% of the non-dairy cattle managers were aware. Dairy farmers mostly sent pregnant cattle to slaughter due to disease or injury, while slaughter based on production-related concerns was also common. However, there may be a link between production and health-related concerns, since poor production was often mentioned as a reason to expect future health problems such as ketosis.

Oestrus-related behaviour was also frequently mentioned as a reason for mating cattle designated for slaughter, in order to avoid injuries when cattle in oestrus jump on other animals or vice versa. Many farmers viewed the sending of pregnant cattle for slaughter as an ethical dilemma between sending a pregnant female for slaughter and facing various welfare-related problems.

The observed prevalence of pregnant cattle at slaughter was very similar to those reported in the 1960s and 1970s [4,5,6], although our estimate for non-dairy cattle was lower than that for extensively farmed beef cattle in Australia (63% [8], and higher than the expert-elicited median estimates provided by the European Food Safety Authority [11], with medians of 16% for dairy and 11% for beef cattle in the European Union (EU). A feasible explanation is that there are many large dairy herds in Denmark compared to other EU countries, and managers in larger herds may be more likely than managers of smaller herds to send a pregnant cow for slaughter. However, the reported European data on which the mentioned estimates are based are sparse and of poor quality. Differences in region and production systems may also influence the prevalence, for example, of cows kept in extensive beef production systems. Reasons for slaughtering cattle were also reported for English famers [7], but these farmers mostly did not know that their cattle were pregnant, unlike most of the Danish farmers in the current study. Consequently, the English farmers reported infertility as the most common reason for slaughter, with mastitis and old age following, while we reported that disease was the most important reason for Danish dairy farmers. These reasons are not comparable, because most Danish dairy farmers knew the pregnancy status and because major changes have occurred in the cattle industry over the last decades (e.g., increased herd size).

Although most dairy farmers knew that their animals were pregnant, a few were surprised to learn this because they had had a negative pregnancy test. Many farmers saw the slaughter of pregnant animals as a problem, but a pregnancy period of more than nine months gives ample time for the occurrence of different events that could lead to disease or injury in modern production systems, and therefore deviation from the planned full lactation of a cow. Involuntary slaughter can result from such incidents, where farmers feel that they must react. This challenge has not previously been addressed in the literature. Management options can include treatment (if possible), slaughter, or on-farm euthanasia. One farmer stated that slaughter was equally as good as euthanasia for a lame cow; so if the cow was suitable for transport, slaughter was chosen. Nevertheless, several farmers mentioned that they did not want to slaughter pregnant cows but still did so (with reference to disease or injuries), which highlights the dilemma that farmers face between keeping the pregnant dam and its foetus and protecting the dam against unnecessary suffering and thereby losing the foetus. In more extensive beef production, the farmers may not have deliberately bred the animals, but merely let nature prevail by leaving the bull and female cattle together. Natural living may also be requested by consumers, and keeping bulls, heifers, and cows apart can be seen as a problem from that perspective. Many farmers demonstrated an awareness of the dilemmas they faced. Many also appeared to have ethically founded reasons for sending pregnant cows for slaughter, although some may not have given it much thought. Farmers were not asked about their attitudes to the practice of raising animals, which are eventually going to be slaughtered, but there is reason to think that they accept this practice without any moral qualms and only have ethical concerns when either animals suffer unnecessarily or are killed without a good reason.

We experienced a very high proportion of responders. Most farmers were willing to talk to us, although some animosity was expressed in the beginning of the interviews. However, this was resolved in most cases, although some farmers did not want to participate. The interviewer (S.S.N.) is known to many farmers from his role in the Danish paratuberculosis programme, and therefore, they may have been fine by being interviewed. We did make every effort to use only publicly available information to get to the farmers though, and we made sure that they at all times could retract their consent.

Based on our findings, it is not possible to claim that the slaughter of pregnant cows and heifers in Danish dairy and non-dairy production occurs without ethical thinking and justification by the farmers. Even though the answers to our survey may contain some elements of self-serving retrospective justification, the overall impression that emerges is that most farmers who knowingly send pregnant cows or heifers for slaughter do so with a “heavy heart”, based on a perceived balance of conflicting concerns.

## Figures and Tables

**Table 1:** Prevalence of pregnant cattle in different stages of gestation, overall and stratified by production type and parity among all the cattle slaughtered at a major Danish abattoir from 16–19 October 2017.

Overall	No. and Prevalence (%) of Pregnant Cattle at Different Gestation Stages								
	N	Overall		>1/3 (93 Days ^1^)		>2/3 (186 Days)		>9/10 (252 Days)	
		No.	%	No.	%	No.	%	No.	%
All animals	814	187	23	131	16	39	5	3	0.4
Dairy cattle									
All animals	583	130	22	90	15	30	5	3	0.5
Parity = 0	79	16	20	4	5	1	1.3	0	0
Parity >0	504	114	23	86	17	29	6	3	0.6
Non-dairy cattle									
All animals	231	57	25	41	18	9	4	0	0
Parity = 0	135	21	16	15	11	1	0.7	0	0
Parity >0	96	36	38	26	27	8	8	0	0

^1^ Based on a gestation length of 281 days.

**Table 2:** Distribution of justifications for the 168 slaughtered pregnant cows for which a reason for sending cattle for slaughter was given by the farmer. ^1^

Overall	>1/3 (93 Days)			95% Confidence Interval
	N	No.	%	
All animals	168	80	48	-
Dairy herds				
All animals	118	56	-	-
Production	51	17	30	20–43%
Health	67	39	70	57–80%
Age	0	0	0	0–6%
Non-dairy herds				
All animals	50	24	-	-
Production	32	15	63	43–79%
Health	13	6	25	12–45%
Age	5	3	13	4–31%

^1^ No information was available for 12 dairy and six non-dairy cattle slaughtered during the period, as the farmers could not be reached or did not want to participate.

## Appendix A

**Table A1:** Comments from 61 dairy cattle farmers sending pregnant animals to slaughter in Denmark in October 2017, sorted by estimated gestation month of the oldest foetus delivered from the herd. Statements in [] are comments from authors. Comments are translated from Danish.

ID	Comments to the Question: What is your attitude towards sending pregnant animals for slaughter?
Second month of gestation (estimated for oldest foetus of the herd)	
1	Deep down, I don’t like sending pregnant animals for slaughter but we have had health problems after mixing animals of different herds, with the introduction of mycoplasma to our herd, so we need to control this disease.
2	Normally, I don’t like sending pregnant animals for slaughter, but they had been with the bull and were not more than two months pregnant, because the bull entered their premises on 15 August [slaughtered 19 October]. Previously, we sent one cow in the very last part of gestation because we thought she was not pregnant. We were fined and learned from that, so now we always examine if in doubt whether they might be at the end of pregnancy.
3	I trust that the authorities put together reasonable rules for me to stick to, so that the animals do not suffer. Normally, we don’t slaughter many pregnant animals, and we should not slaughter animals far into pregnancy, but we had too many animals on the farm compared to the number we were allowed to have.
4	It is a problem if it is close to calving, but non-pregnant animals can cause a lot of unrest, so we try to prevent that. But we slaughter the pregnant [heifer] before it is too late.
Third month of gestation (estimated for oldest foetus of the herd)	
5	Have just taken over the farm, and there was a lot of unrest in the herd—especially among the older cows. Have slaughtered two cows within a short period, probably as a consequence of this unrest; good cows that suddenly lay with broken legs. Therefore, we have started inseminating all cows, including high somatic cell count cows that are heading for slaughter. When they are inseminated, the milk yield decreases and therefore they are culled. Actually, it goes against my previous philosophy, but I prefer to have cattle that do not harm each other.
6	We should avoid sending pregnant animals for slaughter. It is a pity to inseminate a cow and not take advantage of it. Generally, cows are not given a cull code if they have a positive pregnancy diagnosis. I think that the last 10^th^ of gestation is too late and I think it could be tightened.
7	I do not care at all.
8	We do not inseminate cows destined for culling, but you never know what can happen to the rest. If they are diseased, you have to cull them. I haven’t experienced particular problems with unrest, even if they are not inseminated. They are Jerseys, and I have only experienced unrest among the Holsteins.
9	We aim not to slaughter in late gestation, but within the first three to five months there is little concern. We consequently inseminate all animals and subsequently sort. You cannot predict the future of a cow, and she can become diseased or her milk production can cease.
Fourth month of gestation (estimated for oldest foetus of the herd)	
10	In my world, the animals are here to produce milk and calves. But we stick to the law and should make sure it doesn’t get any stricter, because the calves do not suffer.
11	It is against my principles to slaughter pregnant animals, but the somatic cell count went up and down. Even if she produced a lot of milk, we chose slaughter in this case.
12	My mother checks if the rules are kept by going through the papers when I provide her with a list of planned cull cattle. I think that the rules are fair. I don’t think it is fair to the cow to keep her for the entire pregnancy if, for example, she has mastitis. It is better to cull her if she has health problems.
13	In general, I am against sending pregnant animals for slaughter when we are close to calving. I am an organic farmer so I cannot medicate the animals myself after calving, and I was afraid she would need treatment, and that would affect her milk yield.
14	The Danish rules are acceptable, but it will be difficult if the German rules with no culling in the last third of gestation are introduced. It is sufficient that you cannot slaughter in the last month. In nature, if a wolf takes the cow, it doesn’t ask if she is pregnant! We inseminate for two reasons: partly to have calm cows—otherwise, the cows in heat will tear everything apart—and partly to produce a new calf. But a lot of things can happen between insemination and calving: she could suffer an injury and then it is better that she is slaughtered.
15	I probably act like in Germany: one month before dry-off (that is 2/3 into gestation) is the last chance for me to assess whether she can complete the pregnancy. A pregnancy is a tough process. But the first time I assess whether a cow should be culled is prior to insemination.
16	I only slaughter pregnant animals if something is wrong with them, as in the present case, where we couldn’t milk her [the cow had trauma to one teat].
17	When calving is getting too close, then it is a problem, but early in pregnancy it is OK. A non-pregnant animal destined for slaughter can also cause a lot of trouble.
18	I have suckler cows and I would not have sufficient space for her in winter if she became sick. I only cull pregnant animals if they have health problems, and it should be done early as with this one, which was around four months into gestation. But if there is a crisis in a farm, then the banks require us to slaughter cows right to the last legal date [this refers to farms put under administration by a bank due to financial problems]. I have just experienced that, and I don’t like it; a new farmer should be allowed to take over a herd rather than sending it for slaughter.
19	It is okay if it is early in gestation. Normally, I do not slaughter cattle if they are pregnant. It is a bit difficult. But if they have health problems, then it has to be done.
20	It would be better if they weren’t pregnant when sent to slaughter, but we do it if they are sick. Slaughter of cattle up to four weeks before calving—that is too late. We do have problems with restless cows “jumping around” if they are not inseminated, but we just have to live with that.
21	We stick to the law, because we shouldn’t slaughter right up to calving. We should also be able to slaughter a cow with problems that mean she will suffer.
22	We don’t slaughter cows in the last third of gestation: only if they have a negative pregnancy diagnosis. We have had one with a negative pregnancy diagnosis, and this cow turned out to be pregnant and we got fined. Normally we will—if in doubt—have a pregnancy test done before slaughter.
23	I wouldn’t slaughter cows at the end of pregnancy, not even as far into gestation as is allowed. I find it ethically wrong, and I would not risk being fined. If the cows are not far into pregnancy, then it is okay. I inseminate to maintain a calm herd, but then I often cull when they have calved.
24	I don’t feel good about it, but if a cow has problems lasting until the next lactation, then I would rather cull her. It is like choosing between “plague or cholera”.
Fifth month of gestation (estimated for oldest foetus of the herd)	
25	We are expanding, so we keep all the animals we can. But our culled cows produced no milk, weighed 800 kg, and were very large when they were only three months into gestation. I find it extreme that you can slaughter a cow up to 250 days into pregnancy. It should not be more than 200 days. When the foetus can survive, the pregnant cow should not be slaughtered.
26	If they are not too far into pregnancy, then it is not a problem to slaughter. But we avoid sending cows for slaughter far into pregnancy.
27	We never inseminate cows that we plan to slaughter. However, sometimes things happen so we have to slaughter a cow anyway. In the present case, the cow was culled because she was not pregnant, which turned out to not be correct. That is a pity.
28	A few times over the last 10 years we assessed that a cow could not survive calving even if she was close to dry-off. Otherwise, we don’t slaughter cows far into pregnancy. We do not inseminate cows that we do not believe can endure having another calf. Calving is a large burden. I would be annoyed with myself if I were not able to correctly assess whether a cow could endure the burden of giving birth to a calf.
29	This cow was only three months pregnant; I always check. In general, pregnant cows should not be sent for slaughter, but a decision can be changed if there are health problems such as with the legs, the udder, or other problems. We pride ourselves on having high animal welfare and doing the right thing, but it is not always easy when there are dilemmas such as pregnancy versus health.
30	If they are too far into pregnancy (i.e., no later than drying off eight at weeks before calving), then we do not slaughter them.
31	The attitude is that they are not slaughtered if more than halfway through pregnancy. Then, I have to be better at assessing which to cull. However, they are slaughtered if something serious happens. I do not inseminate to have a calmer herd: I don’t like that and I cannot manage it.
32	I don’t like sending pregnant animals for slaughter, only if there is a good reason such as health problems with bad legs. I don’t want to kill a foetus, and we normally make the cull decision before we inseminate for the same reason.
Sixth month of gestation (estimated for oldest foetus of the herd)	
33	It is not something that I normally do: only if there are health problems or milk production has ceased.
34	I prefer not to slaughter pregnant animals unless there is a health problem such as lameness.
35	As long as we are within the legal boundaries, then it is OK when we look at health. It is difficult to manage from a welfare point of view if we cannot slaughter a cow with lameness or mastitis, which are conditions that would otherwise end with euthanasia anyway. Then, it is better to slaughter them early. If your challenge merely is that you have too many animals, then you shouldn’t slaughter the pregnant ones.
36	It is a bit of a dilemma. Generally, we inseminate all the cows, because they would otherwise romp about and we would have too much unrest with injuries as a consequence.
37	I often inseminate to maintain a calmer herd, but it is rare that I cull animals late in gestation. Four weeks before calving is too late. It is better at two months or 12 weeks. Otherwise, we seriously consider whether she has to go. Then, we have to get the veterinarian to write a transport certificate.
38	I can see that one probably shouldn’t do it, but if they have poor legs, then it is okay. Otherwise, you should euthanise on the premises, but then nothing has been gained. Furthermore, at times we have doubts about how far into pregnancy she is, but a pregnancy diagnosis far into gestation can be challenging when assessing the gestation period.
39	We strive not to slaughter animals far into gestation, whereas we pay less attention to those early in gestation. Normally, we do not slaughter in the last half of gestation. If they are to be culled, then they are normally not inseminated. However, in cases with pronounced signs of heat, we may inseminate, but it is not very often.
40	When we slaughter animals, they are almost all pregnant, because we do not know which cows should be culled. It is only at dry-off that we assess whether a cow should stay in the herd. We inseminate all the animals to have a calmer herd. Otherwise, we have 10–15 cows in heat “jumping around”. That results in a lot of injuries.
41	I would prefer to avoid sending pregnant animals for slaughter, but we do not want to keep a cow that is affected by poor health. If it is a cow in good health, then in my view, slaughter in the last third of gestation could be banned.
Seventh month of gestation (estimated for oldest foetus of the herd)	
42	I would prefer to avoid sending pregnant animals for slaughter, but we are a production facility. We do not know what will happen to a cow after she has been inseminated, but we inseminate to keep the production going by producing calves.
43	We have a milk production, and therefore focus on cows producing milk. We prefer to not slaughter pregnant animals.
44	I do not feel good about sending pregnant animals for slaughter. It is a waste, especially if they are 6–7 months into gestation, but you have to take their health into account.
45	We never slaughter cows in the last two months of gestation: it is rarely done once they are dried off, which happens around eight weeks prior to calving. We inseminate all the animals, primarily to maintain a calm herd, but also because a decision to cull a cow can be reversed if she manages the lactation well, while another doesn’t perform as well.
46	We try to avoid sending pregnant animals for slaughter, although it does happen early in pregnancy. I don’t really think it was ideal with this one, which was half a year into pregnancy. Actually, I think 250 days into gestation it is too late to allow slaughter.
47	Normally, we do not send pregnant animals to slaughter unless there is a reason such as with these ones [one was a cow with only two productive teats after recurrent *Escherichia coli* mastitis, and the other produced very limited milk and according to experience would get fat and then diseased]. We avoid restless cows by insemination, but cows destined for culling are not inseminated.
48	The only thing that interests me in this regard is how far they are into pregnancy. Seventy percent of my cows are pregnant, and if we cull them no later than 60–80 days before calving, then it is fine. Every three weeks, the cows in heat “jump around” if they are not pregnant, and that causes unrest and injuries.
49	There must be an extraordinarily good reason such as accidents or injury to slaughter a cow in the last third of gestation—otherwise she should be kept. As a rule, one should keep pregnant animals—that is what it is all about!
50	As a rule, pregnant animals should not be slaughtered, especially outside the limits of the law. We only inseminate those that we don’t plan to cull, but it is possible that they then have a health problem that necessitates their culling. We do not cull pregnant cows just because production is affected or to get a calmer herd, because there are always others that should be culled sooner.
51	We do not slaughter pregnant cows: this was an emergency. We thought it a shame that she should start the next lactation with ketosis, because experience tells us that if she ceases to produce milk early, then she will be a problem cow with ketosis in the next lactation.
52	We try to avoid sending pregnant cows for slaughter—we have had a lot of focus on that in the past three years. We do not inseminate cows that we plan to cull, but in cases of acute disease, it can be necessary to cull them. We are very focused on not culling pregnant animals, and we do not have problems with unrest in the herd—even if they are not inseminated.
53	I don’t like sending animals to slaughter if they are pregnant. If it happens, then they shouldn’t be more than five to six months into gestation. Preferably, it shouldn’t happen at all.
Eighth month of gestation (estimated for oldest foetus of the herd)	
54	When they are far into pregnancy like this one, then it is not good, but early in pregnancy is fine. It is preferable to have a calm herd with pregnant animals, whereas it causes a lot of unrest if they are in heat every third week. Recently, we had to euthanise a cow after slipping following unrest in the herd.
55	It is ethically wrong. I feel bad about it. But if they do not milk well, and there is still a long time until calving, then they are slaughtered. It is not ethically correct, but what should one do?
56	I haven’t given it much thought, but I guess it is acceptable, as long as it is not within the last four weeks before calving.
57	It is a pity to slaughter pregnant animals, but if there are health problems, then the opportunity to cull should be present. Following a discussion with the veterinarian, it was decided that these two should be culled, as it was assessed that they couldn’t endure one more lactation. It is a good slaughter limit we currently have.
58	We generally choose cows for slaughter early in lactation, so they are not inseminated. However, I prefer to be able to cull cows if they turn out to be unable to endure another lactation.
59	I just took over herd management from my previous manager who bought his own farm. I started by culling the animals in the poorest health. They shouldn’t have been inseminated, and I will not inseminate animals that are to be culled, as happened here. It is possible though that some animals are inseminated, and then something happens that makes slaughter a necessity. Unrest within the herd is also possible if there are too many non-pregnant animals that are in heat and injure each other when they “jump around”. However, there is also a cost to insemination, so we rarely inseminate these.
Ninth month of gestation (estimated for oldest foetus of the herd)	
60	We would rather not slaughter pregnant animals, but at times, it can be necessary for reasons related to health or production. Today, we had an emergency slaughter of a pregnant cow with a broken leg.
61	In the first half of gestation it is okay, but no later than that. We send 250–300 females to slaughter each year and all are examined, but it can happen that one is missed. It costs DKK 5000 [around €670 in January 2019] the first time, and DKK 15,000 [around €2000 in January 2019] the second time. The penalty is bigger than gangsters’ “stupidity” fines.

**Table A2:** Comments from 31 non-dairy-producing farmers sending pregnant animals to slaughter in Denmark in October 2017, sorted by estimated gestation month of the oldest foetus delivered from the herd. Statements in [] are comments from authors. Comments are translated from Danish.

ID	Comments to the Question: What is your attitude towards sending pregnant animals for slaughter?
Second month of gestation (estimated for oldest foetus of the herd)	
62	I have beef cattle and the cow in question was with a bull, so in practice it is impossible to avoid cows becoming pregnant. I do everything to avoid sending them for slaughter when more than four to five months into gestation.
63	I have 10 suckler cows. I was surprised when you said she was pregnant, because I did not know. I feel bad about that. I have just bought a new bull, which will be my breeding bull next year. I didn’t think he was old enough this year, but apparently he was.
64	I was almost certain that she was not pregnant because the bull only jumped her three weeks ago. I am very much against sending a pregnant animal for slaughter, so I always avoid it. I will have to take better care. I am very sorry.
65	Normally, I never slaughter pregnant cows, and it was a mistake that it happened.
66	We have suckler cows that are kept with the bull. Therefore, we cannot avoid them becoming pregnant, and they calved not so long ago, so we knew that they couldn’t possibly be more than three to four months into pregnancy. That is not much, and we only cull pregnant cows when necessary, and we cannot avoid them getting pregnant when they are with the bull. We send them for slaughter early in gestation.
67	We have a veal production and a few Hereford cattle. The animals have been with a bull, so we have very little knowledge of whether they are pregnant. But no. 209 was “definitely” in heat on 2 October, and therefore I did not expect her to be pregnant. Normally, heifers and bulls are kept together, and when they are in heat every three weeks, they might get pregnant. Otherwise, they can tear everything apart. We usually do not slaughter the heifers until they are 16 months old, and then they have been in heat many times and some also become pregnant. If we are in doubt if they are pregnant and suspect that they are far into gestation, then we have a pregnancy test done. It is okay to slaughter them if they are not too far into gestation.
68	I have suckler cows and normally no opinions on the subject if it is not too close to calving. Otherwise, we should not slaughter the pregnant animals.
Third month of gestation (estimated for oldest foetus of the herd)	
69	We have veal calves and we do not cull pregnant cows. That is not something one should do—unless there are health problems.
70	I do not send pregnant animals to slaughter—except by mistake or if the animals are suffering. Not animals that are far into gestation. The ones in question had horns and chased the other animals in the herd.
71	The animals are kept with the bull on a daily basis in July, and they will become pregnant. They are mated naturally and if they are not too far into pregnancy, then I don’t see any problems with sending them to slaughter. These two were culled because they were old and heavy—I prefer to have younger ones instead.
72	It is problematic with “summer mastitis” when ear tags are no longer available. It causes problems so that eventually we have to cull a pregnant cow. In general, I think pregnant animals are allowed to be culled too far into gestation, unless they have health problems.
73	The females are with a bull, so they will become pregnant. If it is early like with this one, which was expected to calve in seven months, then it is not a problem. The rules are fine as they are: I find them appropriate.
Fourth month of gestation (estimated for oldest foetus of the herd)	
74	You should not slaughter pregnant animals within the last three, four, or five months of gestation. However, I am puzzled that she was pregnant, because she had only been with her son, which was 1.5 years old, and there were no other bulls with them.
75	I don’t do that!
76	She calved in June and was subsequently with a bull, so she cannot have been far into gestation. Otherwise, we normally do not send pregnant animals for slaughter.
77	We have Hereford cattle and are city people, and as such we don’t think that you should slaughter pregnant animals, except if they have a mechanical injury or practical problems. We have split the animals so they are not bred to the bull, unless we plan to and keep them together. We only use natural mating.
78	It is unfortunate. I did not know she was pregnant. We have suckling cows and she was with a bull, but had I known she was pregnant then she might have stayed. It is not fair to slaughter them just before calving, unless there are problems.
79	I have beef cattle and they are with a bull, at least for a period. Avoiding pregnancy is not easy, and it is not easy to see if they are pregnant because they don’t develop udders like dairy cows, but normally I can see if they are far into gestation. Four weeks prior to calving may be a bit close, because you shouldn’t slaughter cattle far into gestation. A few months before calving is better, and some animals have to go, so we have to make sure that it doesn’t get too close. It also results in calmer animals if there are not too many in heat. I am 83 years old and it shouldn’t be like in the old days, where the sow gave birth in the slaughter house. That was a mess.
Fifth month of gestation (estimated for oldest foetus of the herd)	
80	I don’t have a particular opinion on this, when we keep within the law. We send them for slaughter well in advance of calving. We only have six to eight cows and they are with the bull, so they will become pregnant. The bull only arrived in January.
81	She was a “bulling” cow [masculinised due to cystic ovaries according to the farmer, i.e., cystic ovarian disease]. I thought she was pregnant, but wasn’t sure. However, when the animals are kept together it is impossible to keep the cows and bulls apart. It is also easier to have growth when they are pregnant. It leads to better body condition and pregnant animals are also calmer. They can be very restless when in heat.
82	We have a suckling herd where the bull is kept with the others. He is separated from the rest at a specific point in time, and I did not expect her to be pregnant because the other cows jumped her. We do not examine them systematically. I think that one month before calving is the very latest that a cow should be sent off for slaughter, and I will normally not do it in the last half year of pregnancy.
83	Sending pregnant cattle to slaughter three, four, or five weeks before calving is too close, and I would definitely support stricter rules. The last third of gestation like in Germany sounds like a possibility. Our animals are with a bull in a meadow during summer and they become pregnant. If a cow should be culled, you should avoid getting her pregnant, but it is difficult in a production where the cow is kept with the bull.
84	My attitude is that you should not slaughter pregnant animals. I took over the herd on 1 July and did not have any influence on which cows had been with a bull. I thought it was a pity to leave her with the other cows because she was afraid of them.
85	I prefer not to slaughter pregnant animals. Cull cows shouldn’t be with a bull, but in principle, the breeding animals are always with a bull. We only have around 10 animals.
86	I don’t have cows, but a veal production. I prefer not to slaughter pregnant animals, but sometimes the animals mix. If the heifer is in the last month of gestation then I prefer to have the calf.
87	Our animals are with a bull to keep them calmer. Therefore, they also become pregnant. Our animals are used for nature conservation, and if there is no bull with the herd, then they become restless when in heat. We also have a herd without a bull, and there are often problems when they are in heat. It would be problematic for Viking [a Danish breeding company] to make a specific pregnancy diagnosis late in pregnancy if the limit is set to the last third of gestation.
88	In general, it is not good to cull pregnant animals, but four to five months into gestation cannot be a problem. If they are more than five months into gestation, then we keep them and deliver the calf before the cow is slaughtered. We had a breeding bull that was removed in May. Next year, those destined for slaughter should not be with a bull.
Sixth month of gestation (estimated for oldest foetus of the herd)	
89	It seems foolish in a beef production to slaughter pregnant animals. We make a living from the calves. If it is early in gestation, then it can be difficult to know that they are pregnant, and I didn’t know in the present case.
Seventh month of gestation (estimated for oldest foetus of the herd)	
90	I don’t support that—it really is a pity. I have suckling cows that are normally with a bull. I thought the old bull was sterile, but I got five calves with the new bull, which was not with this cow. He cannot have been sterile then. It is a pity!
91	I have suckling cows and they are with a bull. It is not possible to have a herd with a bull and then manage which ones become pregnant. But culling depends on the stage of gestation—if there are only a few months left of gestation, then it is too far. But we should also be able to be in the shed, and it causes a lot of unrest if they are in heat every third week. In the present case, they had just been removed from their calves, which were six to seven months old, and therefore they could not be in the end of gestation.
Eighth month of gestation (estimated for oldest foetus of the herd)	
92	Pregnant animals should not be slaughtered, and I would like to apologise that she was sent to slaughter. She had poor legs, and I went to check the rules. I assessed that it was best to send her to slaughter according to the rules we currently have. This never happened before, and normally I only inseminate animals that I intend to keep. It was the intention to keep her another five to seven years.
